# Supplementary material for: Effects of parental migration on early childhood development of left-behind children in Bangladesh: Evidence from a nationally representative survey
Source: PLoS One. 2023 Nov 30;18(11):e0287828. doi: 10.1371/journal.pone.0287828 (PMC10688621; doi:10.1371/journal.pone.0287828)
Supplement: S1 Table — (DOCX) [file pone.0287828.s001.docx]

**Table S1:** Effects of father migration on early childhood development

| **Basic characteristics** | **Early childhood development Index (ECDI)** | | |
| --- | --- | --- | --- |
|  | **Odds Ratio** | **95% Confidence Interval** | |
| **Father’s Migration** | | | |
| Not migrated | Ref |  | |
| Abroad | 0.74** | 0.54-0.93 | |
| Within country | 0.97 | 0.80-1.18 | |
| **Child age** | | | |
| 3 years | Ref |  | |
| 4 years | 1.85** | 1.74-1.98 | |
| **Sex of child** | | | |
| Male | Ref |  | |
| Female | 1.00 | 0.94-1.06 | |
| **Residence** | | | |
| Urban | Ref |  | |
| Rural | 1.08 | 0.98-1.18 | |
| **Division** | | | |
| Barishal | Ref | |  |
| Chattogram | 0.92 | | 0.81-1.04 |
| Dhaka | 0.46** | | 0.40-0.53 |
| Khulna | 0.65** | | 0.57-0.76 |
| Mymensingh | 0.79** | | 0.67-0.93 |
| Rajshahi | 0.74** | | 0.63-0.85 |
| Rangpur | 0.50** | | 0.43-0.58 |
| Sylhet | 0.88 | | 0.76-1.03 |
| **Mother’s education** | | | |
| Pre-primary or none | Ref | |  |
| Primary | 0.97 | | 0.87-1.09 |
| Secondary | 1.17** | | 1.06-1.30 |
| Higher secondary+ | 1.44** | | 1.25-1.66 |
| **Mother's functional difficulties** | | | |
| Has functional difficulty | Ref | |  |
| Has no functional difficulty | 0.49** | | 0.39-0.62 |
| No information | 0.68* | | 0.48-0.95 |
| **Wealth index quintile** | | | |
| Poorest | Ref | |  |
| Second | 1.15** | | 1.04-1.27 |
| Middle | 1.25** | | 1.12-1.39 |
| Fourth | 1.53** | | 1.37-1.71 |
| Richest | 1.83** | | 1.60-2.09 |

**Notes:** ^**^p<0.05^, *^p<0.01
