# Supplementary material for: Effects of parental migration on early childhood development of left-behind children in Bangladesh: Evidence from a nationally representative survey
Source: PLoS One. 2023 Nov 30;18(11):e0287828. doi: 10.1371/journal.pone.0287828 (PMC10688621; doi:10.1371/journal.pone.0287828)
Supplement: S2 Table — (DOCX) [file pone.0287828.s002.docx]

**Table S2:** Effects of mother’s migration on early childhood development, Bangladesh

| **Basic characteristics** | **Early childhood development Index (ECDI)** | | |
| --- | --- | --- | --- |
|  | **Odds Ratio** | | **95% Confidence Interval** |
| **Mother’s Migration** | | | |
| Not migrated | Ref | |  |
| Abroad | 1.51 | | 0.82-2.75 |
| Within country | 0.80 | | 0.61-1.05 |
| **Child age** | | | |
| Age 3 years | Ref | |  |
| Age 4 years | 1.54** | | 1.43-1.65 |
| **Sex of child** | | | |
| Male | Ref | |  |
| Female | 1.00 | | 0.94-1.07 |
| **Residence** | | | |
| Urban | Ref | |  |
| Rural | 1.07 | | 0.97-1.17 |
| **Region** | | | |
| Barishal | Ref |  | |
| Chattogram | 0.89 | 0.78-1.01 | |
| Dhaka | 0.45** | 0.39-0.51 | |
| Khulna | 0.66** | 0.57-0.76 | |
| Mymensingh | 0.78** | 0.66-0.92 | |
| Rajshahi | 0.74** | 0.64-0.86 | |
| Rangpur | 0.50** | 0.43-0.58 | |
| Sylhet | 0.88 | 0.75-1.02 | |
| **Attendance to early childhood education** | | | |
| No | Ref |  | |
| Yes | 2.08** | 1.91-2.26 | |
| **Mother’s education** | | | |
| Pre-primary or none | Ref |  | |
| Primary | 0.95 | 0.85-1.05 | |
| Secondary | 1.11* | 1.00-1.24 | |
| Higher secondary+ | 1.33** | 1.15-1.54 | |
| **Mother's functional difficulties** | | | |
| Has functional difficulty | Ref |  | |
| Has no functional difficulty | 0.49** | 0.39-0.62 | |
| No information | 0.67* | 0.47-0.96 | |
| **Wealth index quintile** | | | |
| Poorest | Ref |  | |
| Second | 1.15** | 1.04-1.28 | |
| Middle | 1.22** | 1.10-1.36 | |
| Fourth | 1.47** | 1.31-1.64 | |
| Richest | 1.71** | 1.50-1.94 | |

**Notes:** ^**^p<0.05^, *^p<0.01
