# Supplementary material for: Effects of parental migration on early childhood development of left-behind children in Bangladesh: Evidence from a nationally representative survey
Source: PLoS One. 2023 Nov 30;18(11):e0287828. doi: 10.1371/journal.pone.0287828 (PMC10688621; doi:10.1371/journal.pone.0287828)
Supplement: S3 Table — (DOCX) [file pone.0287828.s003.docx]

**Table S3:** Effects of migrating both parents on early childhood development, Bangladesh.

| **Basic characteristics** | **Early childhood development Index (ECDI)** | | |
| --- | --- | --- | --- |
|  | **Odds Ratio** | | **95% Confidence Interval** |
| **Both Parents’ Migration** | | | |
| Migrants none of parents | Ref | |  |
| Migrants both parents | 0.63** | | 0.48-0.97 |
| **Child age** | | | |
| 3 years | Ref | |  |
| 4 years | 1.54** | | 1.43-1.67 |
| **Sex of child** | | | |
| Male | Ref | |  |
| Female | 1.00 | | 0.93-1.08 |
| **Residence** | | | |
| Urban | Ref | |  |
| Rural | 1.12* | | 1.01-1.24 |
| **Division** | | | |
| Barishal | | Ref |  |
| Chattogram | | 0.93 | 0.81-1.06 |
| Dhaka | | 0.45** | 0.39-0.53 |
| Khulna | | 0.65** | 0.56-0.75 |
| Mymensingh | | 0.77** | 0.64-0.92 |
| Rajshahi | | 0.73** | 0.62-0.85 |
| Rangpur | | 0.50** | 0.43-0.59 |
| Sylhet | | 0.86 | 0.74-1.01 |
| **Attendance to early childhood education** | | | |
| No | | Ref |  |
| Yes | | 2.05** | 1.87-2.24 |
| **Mother’s education** | | | |
| Pre-primary or none | | Ref |  |
| Primary | | 0.92 | 0.83-1.03 |
| Secondary | | 1.08 | 0.97-1.21 |
| Higher secondary+ | | 1.25** | 1.07-1.46 |
| **Mother's functional difficulties** | | | |
| Has functional difficulty | | Ref |  |
| Has no functional difficulty | | 0.49** | 0.39-0.62 |
| No information | | 0.69 | 0.47-1.01 |
| **Wealth index quintile** | | | |
| Poorest | | Ref |  |
| Second | | 1.14** | 1.03-1.27 |
| Middle | | 1.24** | 1.12-1.39 |
| Fourth | | 1.54** | 1.37-1.74 |
| Richest | | 1.85** | 1.61-2.14 |

**Notes:** ^**^p<0.05^, *^p<0.01
