# Supplementary material for: Effects of parental migration on early childhood development of left-behind children in Bangladesh: Evidence from a nationally representative survey
Source: PLoS One. 2023 Nov 30;18(11):e0287828. doi: 10.1371/journal.pone.0287828 (PMC10688621; doi:10.1371/journal.pone.0287828)
Supplement: S4 Table — (DOCX) [file pone.0287828.s004.docx]

**Supplementary Table 4:** Effects of migrating one of parents on early childhood development index, Bangladesh.

| **Basic characteristics** | **Early childhood development Index (ECDI)** | |
| --- | --- | --- |
|  | **Odds Ratio** | **95% Confidence Interval** |
| **Any one of Parent’s Migration** | | |
| Migrate none of parents | Ref |  |
| Migrant one of parents | 0.85** | 0.77-0.95 |
| **Child age** | | |
| 3 years | Ref |  |
| 4 years | 1.54 | 1.44-1.65 |
| **Sex of child** | | |
| Male | Ref |  |
| Female | 1.00 | 0.94-1.07 |
| **Residence** | | |
| Urban | Ref |  |
| Rural | 1.08 | 0.98-1.19 |
| **Division** | | |
| Barishal | Ref |  |
| Chattogram | 0.90 | 0.79-1.02 |
| Dhaka | 0.45** | 0.39-0.52 |
| Khulna | 0.65** | 0.56-0.75 |
| Mymensingh | 0.78** | 0.66-0.93 |
| Rajshahi | 0.74** | 0.63-0.86 |
| Rangpur | 0.49** | 0.42-0.57 |
| Sylhet | 0.88 | 0.75-1.02 |
| **Attendance to early childhood education** | | |
| No | Ref |  |
| Yes | 2.07** | 1.90-2.26 |
| **Mother’s education** | | |
| Pre-primary or none | Ref |  |
| Primary | 0.95 | 0.85-1.06 |
| Secondary | 1.12* | 1.01-1.25 |
| Higher secondary+ | 1.33** | 1.16-1.54 |
| **Mother's functional difficulties** | | |
| Has functional difficulty | Ref |  |
| Has no functional difficulty | 0.50** | 0.39-0.63 |
| No information | 0.69* | 0.50-0.96 |
| **Wealth index quintile** | | |
| Poorest | Ref |  |
| Second | 1.16** | 1.05-1.28 |
| Middle | 1.24** | 1.12-1.38 |
| Fourth | 1.50** | 1.34-1.68 |
| Richest | 1.76** | 1.54-2.00 |

**Notes:** ^**^p<0.05^, *^p<0.01
