# Supplementary material for: Effects of parental migration on early childhood development of left-behind children in Bangladesh: Evidence from a nationally representative survey
Source: PLoS One. 2023 Nov 30;18(11):e0287828. doi: 10.1371/journal.pone.0287828 (PMC10688621; doi:10.1371/journal.pone.0287828)
Supplement: Attachment — Submitted filename: Response to the Reviewers.docx [file pone.0287828.s006.docx]

| Reviewers’ comments | Authors’ response |
| --- | --- |
| Academic editor |  |
| Situate/relate this study in relation to earlier similar studies, especially in the discussion. | Relevant studies in LMIC settings are scarce, and studies conducted in high-income countries are not directly comparable to our study. However, we have revisited the relevant literature and updated the corresponding sections accordingly. |
| Ensure that the reference style of the journal is adhered to all through the manuscript. | We have updated reference list as per the journal’s style. |
| Reviewer#1 |  |
| This study examine the influence of parental migration on child welfare and early development outcomes in Bangladesh. The study uses a large sample which is one of the strengths of this paper. The findings especially around the impact of both parents migration on child development is worthy of dissemination to a wider audiences and specialist researchers, policy practitioners, CSOs engaged in child welfare and development. The findings have relevance to other low and middle income countries. I only have minor comments as I feel this study is clearly written the approach has been adequately described and can be easily followed by readers. | Many thanks to minutely read our paper. We have addressed your comments and provided a point-by-point response below. |
| a)I would advise the authors to closely follow the citation style of the journal especially citation in text. | We have revisited to all citations and ensured all citations presented in journal’s tyle. |
| Introduction |  |
| b) The authors zoom a little too quickly to Bangladesh even when a 'funnel approach' would help readers set the context for this paper. The 'funnel approach' is setting the global scene, a regional perspective and finally anchoring the study in Bangladesh. | We have now edited the relevant paragraph to address your comment. The relevant edited paragraph of the introduction section now reads (page 4)-  *An estimated 244 million children are left behind (defined as individuals under 18 years of age whose parents have relocated to another place for a period exceeding six months) globally, around 163 million of them live in low- and middle-income countries (LMICs) [1, 2]. The Asia-Pacific region, including Bangladesh with an estimated 6 million left-behind children, has the highest number of such children, totalling an estimated 118 million, followed by sub-Saharan Africa (33 million) and Latin America (12 million) [2]. The primary reason for this migration*, *which results in children being left behind, is that parents frequently embark on migration journeys in pursuit of enhanced economic prospects [3, 4]. Left behind children confront a multitude of challenges stemming from their separation from parents, social isolation, and economic disadvantage [1, 5]. While migration can yield economic benefits for families remaining in the home country, the aforementioned difficulties can profoundly impact the cognitive, socio-emotional, and physical* *development of these children [4].* |
| Methods |  |
| c) Was this a secondary data analysis or did you use primary data. This is not perfectly clear and is a little ambiguous. I would be clearer in bringing this out. | We have now clarified this issue in the study setting and data section under the methods section. The relevant sentences now read (page 5)-  *We conducted an analysis of secondary data derived from the 2019 Multiple Indicator Cluster Survey (MICS) in Bangladesh. This comprehensive survey took place from January to June 2019.* |
| d) Have similar data sets been utilized in other countries? It would help to reference them here if this is the case. | We have added the following sentence to address your comment (page 5).  *It is worth noting that the MICS is a household survey developed by UNICEF, conducted in various countries, with the aim of providing statistically rigorous data on a wide range of indicators concerning the situation of children and women.* |
| Results |  |
| I would use 'demographic' characteristics instead of 'background' statistics. | We have addressed your comment though editing relevant sentences. Please check the result section. |
| Discussion |  |
| I would do more to link this study with previous efforts. The reference list alone betrays this deficiency. | There are few studies available in our specific area of research, and we have diligently cited and compared all relevant research findings with our study. We have reviewed the reference list once more and made necessary updates to it. |
| Overall, this appears to have been a competently conducted study and would recommend publication after minor revisions | Thank you very much for your thorough review of our paper and for providing valuable comments. |
| Reviewer#2 |  |
| Thank you for the opportunity to review this manuscript which focuses on the effect of parental migration on early childhood development in Bangladesh. The importance of studying this subject at individual country level is aptly stated by the authors. The manuscript is generally well written bringing out the objectives, results, and conclusion of the research concisely. The subject matter is a topical issue which will be of interest to both clinical and non-clinical specialties in different countries. | Many thanks for your useful comments. We have addressed them all and provided point-by-point response below. |
| Minor issues |  |
| 1. The authors should revise the language and syntax to improve readability e.g., “on-track status” could be inserted after “were indicators” in line 4, page 12. The discussion section also contains some the language and syntax issues for e.g., line 4, page 22. | The relevant sentences have been edited to address this issue. Furthermore, we have thoroughly reviewed the entire manuscript to ensure that no such errors are present in the text. |
| 2. “Bangladesh” does not need to reflect in the title of Table 1. | We have made the necessary corrections to address your comment. |
| 3. The results in Table 2 suggest that the first row ought to be captioned as “Children who are developmentally on track for indicated domains”. The authors may however clarify if this is not the intended message to readers. | We have made the necessary corrections to address your comment. |
| 4. Reference 29 should be better cited for reproducibility. | We have updated the suggested reference. |
| Major issues |  |
| 1. The authors should clarify how the sample size of 8,833 was obtained out of the total 23,099 eligible children to avoid confusion. | We have revised the analytical sample section to address your comment. This section now reads (page 5-6)-  *We have included children aged 3-4 years old from whom the ECDI-related questions were asked, following the guidelines provided by UNICEF. Out of the total 23,099 children included in the main survey, there were 8,833 children aged 3-4 years old, with 4,513 children aged 3 years and 4,320 children aged 4 years. Therefore, they were included in the analysis.* |
| 2. The authors stated that “We analyzed a total of 8,833 children, with 4,513 children aged 3 years (24-36 months, N=4,513) and 4,320 children aged 4 years (37-48 months, N=4,320).” For clarity, the authors should re-write this age bracket to avoid the confusion that may result from interpreting 24 months as 3 years or 37 months as 4 years. | We have edited relevant sentences to address this issues. All figures are now presented in year. |
| 3. The ages of the children studied should be consistently stated in the manuscript for e.g., the Conclusion section reports that children 3 – 4 years were studied in contrast to 2 – 4 years reported in the Methodology section. | We have addressed this issue by editing relevant sentences throughout the manuscript. |
| 4. The result on the association between parental migration and ECD is that children with fathers who migrated abroad had significantly decreased odds of being developmentally on track in the overall ECDI (OR: 0.74, 95% CI: 0.54-0.93). It is however stated differently in the abstract section as a decrease in the odds of a poor ECD. This difference should be reconciled. | Many thanks to bring this in our attention. We have now addressed this issue though adding the relevant sentences. This now reads (page 2)-  *Regarding the association between parental migration and ECDI outcomes, we observed a 26% decrease in the likelihood of overall ECDI among children with a migrated father (OR: 0.74, 95% CI: 0.54-0.93).* |
| Overall, the manuscript is presented in a clear and logical manner that keeps the reader interested. | We thank the reviewer to minutely read our paper. |
