# Supplementary material for: Effects of parental migration on early childhood development of left-behind children in Bangladesh: Evidence from a nationally representative survey
Source: PLoS One. 2023 Nov 30;18(11):e0287828. doi: 10.1371/journal.pone.0287828 (PMC10688621; doi:10.1371/journal.pone.0287828)
Supplement: Attachment — Submitted filename: Response to the Reviewers.docx [file pone.0287828.s007.docx]

| Reviewers’ comments | Authors’ response |
| --- | --- |
| Academic editor |  |
| Background: The beginning of the 3rd and 4th sentences in 2nd paragraph should be rephrased.  The word 'however' at the beginning of both sentences is distorting the flow of the paragraph. | Many thanks to bring this in our attention. We have now edited the relevant sentences. The revised sentences now read-  *………………..However, left-behind children may be at a disadvantage in terms of ECD due to a range of factors such as inadequate care, poor nutrition, lack of stimulation, and social isolation [4, 8]. Yet, research exploring the impact of parental migration on ECD outcomes among left-behind children in LMICs, and particularly in the context of Bangladesh, remains limited………………………………………..* |
| Results: There is disparity in the percentage of children from the rural area in the text and table 1. | Many thanks to bring this typo in our attention. We have corrected the text and the relevant sentence reads-  *…………….Moreover, approximately four-fifths of the children lived in rural areas, and Chattogram division was reported as the region of residence by a similar proportion of the children………………………….* |
